# PEGylated self-assembled enzyme-responsive nanoparticles for effective targeted therapy against lung tumors

**DOI:** 10.1186/s12951-018-0384-8

**Published:** 2018-07-16

**Authors:** Fangyuan Guo, Jiangqing Wu, Wenchao Wu, Dongxue Huang, Qinying Yan, Qingliang Yang, Ying Gao, Gensheng Yang

**Affiliations:** College of Pharmaceutical Science, Zhejiang University of Technology, #18 Chaowang Road, Hangzhou, 310032 People’s Republic of China

**Keywords:** Enzyme-responsive nanoparticles, Cell-penetrating peptide, Matrix metalloproteinase, Cellular uptake, Tumor extracellular environment

## Abstract

**Background:**

Matrix-metalloproteinases, which are overexpressed in many types of cancer, can be applied to improve the bioavailability of chemotherapeutic drugs and guide therapeutic targeting. Thus, we aimed to develop enzyme-responsive nanoparticles based on a functionalized copolymer (mPEG-Peptide-PCL), which was sensitive to matrix metalloproteinase, as smart drug vesicles for enhanced biological specificity and reduced side effects.

**Results:**

The rate of in vitro curcumin (Cur) release from Cur-P-NPs was not markedly accelerated in weakly acidic tumor microenvironment, indicating a stable intracellular concentration and a consistent therapeutic effect. Meanwhile, P-NPs and Cur-P-NPs displayed prominent biocompatibility, biostability, and inhibition efficiency in tumor cells. In addition, Cur-P-NPs showed higher fluorescence intensity than Cur-NPs in tumor cells, implying enhanced cell permeability and targeting ability. Moreover, the internalization and intracellular transport of Cur-P-NPs were mainly via macropinocytosis. Studies of pharmacodynamics and cellular uptake in vitro and biodistribution in vivo demonstrated that Cur-P-NPs had stronger target efficiency and therapeutic effect than Cur-DMSO and Cur-NPs in tumor tissue.

**Conclusion:**

Results indicate that Cur-P-NPs can be employed for active targeted drug delivery in cancer treatment and other biomedical applications.

**Electronic supplementary material:**

The online version of this article (10.1186/s12951-018-0384-8) contains supplementary material, which is available to authorized users.

## Background

Lung cancer is currently one of the most prevalent cancers in the world with high morbidity and mortality [1–3]. In developing countries, in particular, the increasing incidence of lung cancer has not been effectively controlled [4, 5]. Histologically, lung cancer is classified as small-cell lung cancer (SCLC) and non-small cell lung cancer (NSCLC). Due to the high prevalence of tobacco consumption, NSCLC is found to occur more commonly compared to SCLC [6, 7]. Several researchers have reported that neoangiogenesis is a significantly negative prognostic factor in lung cancer [8, 9]. Therefore, an effective anti-angiogenesis drug for NSCLC therapy is urgently required. Curcumin (Cur), an active substance extracted from turmeric, has been widely studied for its anti-inflammatory [10], anti-angiogenic [11], antioxidant [12], wound healing [13, 14], and anti-cancer effects [15]. Furthermore, Cur can reverse chemo-resistance by inhibiting multiple signaling pathways. However, its poor water solubility, structural instability, low membrane permeability, and bioavailability has greatly inhibited the application of Cur in clinic.

In the last two decades, nanotechnology has been greatly developed in the arena of biomedicine, pharmaceuticals, and drug delivery [16, 17]. Nanoparticles, one of the representative nano delivery systems, are developed for use in oncotherapy due to specific properties, such as surface modification, good stability, and low toxicity [18]. However, indistinctive tumor cell specific uptake and the rapid elimination of nanoparticles by the reticuloendothelial system (RES) are still barriers in efficient drug delivery in vivo [19]. To address these challenges, functionalized nanoparticles such as magnetic nanoparticles [20–22], redox-sensitive nanoparticles [23], pH-response nanoparticles [24, 25], and enzyme-response nanoparticles [26–28] were designed. Because specific enzymes are overexpressed in tumor cells, enzyme-responsive nanoparticles can be an excellent candidate for designing a smart drug delivery system. Matrix metalloproteinases (MMPs) are over-expressed in many types of cancer. Therefore, they are logical targets for enzyme-triggered therapeutics. MMP targeting peptides have been successfully designed [29, 30]. In addition, increasing evidence demonstrates that cell penetrating peptides (CPPs) can help to enhance the ability of cell penetration in cargo delivery. Therefore, the combination of targeting peptides and CPPs could be a potential strategy for improving the effectiveness of cancer therapy.

In this study, we successfully synthesized a novel enzyme-responsive nanoparticle based on a tri-block biomaterial (mPEG-Peptide-PCL), which reconfigures in response to MMPs that are active and overexpressed in cancers, to guide therapeutic targeting. In mPEG-Peptide-PCL, PCL was used for drug loading; (ACP)-GPLGIAGQr9-(ACP) was selected as the targeting peptide. GPLGIAGQ was designed for degradation by MMP-2 [31], and the exposed cell penetrating peptide r9 would enhance the cellular uptake of nanoparticles [32]; PEGylation could improve the stability of the carrier, and prolong the retention time in vivo. Thereafter, the nanoparticle was prepared by the solvent evaporation method. In vitro, the accumulative releasing rate of the drug (Cur as the model drug) in different pH conditions was also evaluated. Furthermore, the toxicity and cellular uptake (including mechanism study) of the biomaterials were assessed in L929 mouse embryonic fibroblasts and NSCLC A549 cells. In vivo, due to a strong fluorescence effect of Cur, the selective targeting behavior and biodistribution of mPEG-Peptide-PCL nanoparticles were measured using an in vivo imaging system.

## Methods

### Materials

Cur was purchased from Hangzhou Guang Lin Biological Pharmaceutical Co. Ltd. (Hangzhou, China). (ACP)-GPLGIAGQr9-(ACP) was from ChinaPeptides Co. Ltd. (Shanghai, China). Poloxamer188 was obtained from BASF (Shanghai, China). Stannous 2-ethylhexanoate [Sn(Oct)_2_] was obtained from Sigma (St. Louis, MO, USA). Dialysis bags (MWCO = 14,000) were obtained from Gene Star Co. (Shanghai, China). L929 mouse embryonic fibroblasts and A549 cells were from the Cell Bank of the Chinese Academy of Sciences (Beijing, China). Kunming mice were obtained from Zhejiang Academy of Medical Sciences (Hangzhou, China). mPEG (Mn = 1900) and ε-caprolactone (ε-CL) were purchased from Aladdin Chemicals (Shanghai, China). Other reagents (analytical or chromatographic grade) were obtained from Aladdin Chemicals.

### Synthesis of tri-block copolymer

**Fig. 1:**
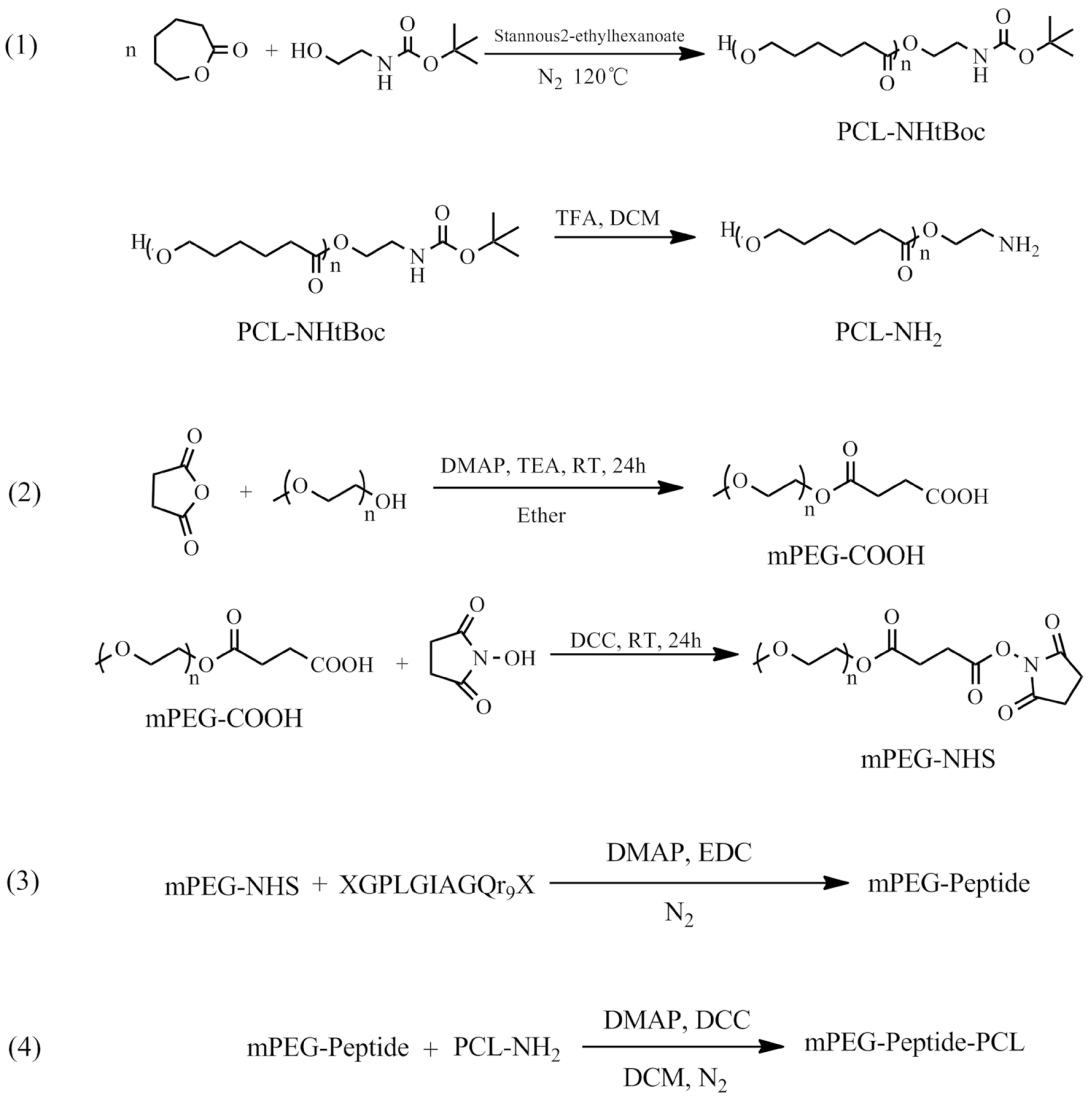
Reaction scheme for elastomer preparation

mPEG-Peptide-PCL was prepared using the segmented synthetic way with mPEG-NHS, peptide and PCL-NH_2_ as different sections. The detailed process has been shown in Fig. 1.

### Synthesis of PCL-NH_2_

2-(tert-Butoxycarbonylamino)-1-ethanol (922.1 mg, 5.72 mmol), ε-caprolactone (9.9987 g, 87.6 mmol), and Sn(Oct)_2_ (catalyst, 40 μL, 0.124 mmol) were mixed in a 100 mL three-necked flask. Then a polymerization reaction was performed at 120 °C under dry nitrogen for 24 h. PCL-NHtBoc was purified by precipitation in cold methanol to remove the unreacted monomer and oligomer, and the purified product was obtained after filtration and vacuum drying at 40 °C for 24 h.

PCL-NHtBoc (5 g) was dissolved into DCM (20 mL), and 4 mL trifluoroacetic acid was added to the solution; the solution was stirred for 8 h at 0 °C under dry nitrogen. After the reaction was completed, the crude copolymer (PCL-NH_2_) solution was washed with saturated NaHCO_3_ solution and distilled water; the extraction process was repeated three times. DCM solution was collected and added drop-wise to cold methanol (1:15, v/v). PCL-NH_2_ was purified by precipitation, and obtained after filtration and vacuum drying at 40 °C for 24 h. The number average molecular weight (Mn), the weight-average molecular weight (Mw), and polydispersity index (PDI) of PCL-NH_2_ were measured by Gel Permeation Chromatography (GPC) analysis [33].

### Synthesis of mPEG-NHS

mPEG (Mw: 1900, 7.6 g, 4 mmol) and butanedioic anhydride (0.8 g, 8 mmol) were dissolved in pyridine (60 mL), then 4-dimethylaminopyridine (DMAP, 73.3 mg, 0.6 mmol) and triethylamine (404.8 mg, 4 mmol) were added, followed by mixing of the solutions. The mixture was stirred under dry nitrogen at room temperature for 24 h. After the reaction was completed, the crude product was dissolved in DCM (20 mL) and precipitated into cold ethyl ether (1:15, v/v). The precipitate (mPEG-COOH) was dried in vacuo at 25 °C for 48 h to remove the solvent.

mPEG-COOH (5.0 g, 2.5 mmol) and N-hydroxy succinimide (1.2 g, 10 mmol) were dissolved in acetonitrile (60 mL), followed by addition of *N*,*N*′-dicyclohexylcarbodiimide (DCC, 1.0316 g, 5 mmol). The reaction was carried out under N_2_ protection at room temperature for 24 h. The purification process used was the same as that used for mPEG-COOH.

### Synthesis of mPEG-Peptide-PCL

The peptide (50 mg, 0.0213 mmol), 1-ethyl-3-(3-dimethylaminopropyl)-carbodiimide hydrochloride (EDC, 27.3 mg, 0.142 mmol), and DMAP (17.4 mg, 0.142 mmol) were dissolved in acetonitrile–water solution (20/80, 10 mL), and the mixture was stirred under dry nitrogen at 0 °C for 2 h to activate the peptide. Thereafter, mPEG-NHS (49.9 mg, 0.0178 mmol) was mixed into the reaction solution, and transesterification was allowed to occur at room temperature for 24 h. The mPEG-Peptide solution was purified by dialysis (Mw: 3500) for 72 h, and lyophilized to the powder form. The Mn, Mw, and PDI were measured by GPC [33].

mPEG-Peptide (100 mg, 0.0194 mmol), DCC (40 mg, 0.1942 mmol), and *N*-hydroxy succinimide (23.7 mg, 0.1942 mmol) were dissolved in DCM (10 mL), followed by stirring of the mixture under dry nitrogen at 0 °C for 2 h. Later, PCL-NH_2_ (165 mg, 0.0291 mmol) was added to the mixture, and the mixture was further stirred at room temperature for 96 h. After the reaction was completed, the mPEG-Peptide-PCL solution was purified by dialysis (Mw: 7000) for 72 h, and lyophilized to powder. The structure of mPEG-Peptide-PCL was confirmed by Fourier transform infrared (FT-IR) spectroscopy (Nicolet 6700; Thermo Fisher Scientific, USA) and ^1^H-NMR spectroscopy, and the Mn, Mw, and PDI of mPEG-Peptide-PCL were measured by GPC [33]. Meanwhile, mPEG-PCL as a contrast compound was aggregated by mPEG and ε-caprolactone as per the method described in “Synthesis of PCL-NH_2_” section.

### Preparation of Cur-loaded NPs

Cur-P-NPs were prepared by the emulsion-solvent evaporation method. The optimal conditions for the preparation were as follows: Cur (2.4 mg) and mPEG-Peptide-PCL (24.0 mg) were co-dissolved in 2 mL acetone, and added drop-wise to 10 mL of an aqueous phase containing Poloxamer-188 (20.0 mg) under magnetic stirring. The mixture was stirred for 4 h to remove acetone. The NP suspension was then filtrated through 0.45 μm filter membrane to remove the unwrapped Cur and achieve a homogeneous suspension. At last, the nanoscaled suspensions were centrifuged at 19,000 rpm for 30 min. The precipitate was collected and washed twice with deionized water, and lyophilized. The fundamental characterization of particle size, polymer dispersity index (PDI), and Zeta potential were measured by a laser particle analyzer (Malvern Zetasizer Nano-ZS90; Malvern, UK).

### Entrapment efficiency (EE) and drug loading (DL)

The drug content of Cur-P-NPs was determined by ultraviolet spectrophotometry with a detection wavelength of 420 nm. Drug entrapment efficiency (EE%) and drug-loading (DL%) content were calculated based on a calibration curve. The equations used were as follows:

1 $${\text{EE}\%}\, = \,\frac{\text{Weight of drug in nanoparticles}}{\text{Weight of feed drug}}\, \times \,100$$

2 $${\text{DL}\%}\, = \,\frac{\text{Weight of drug in nanoparticles}}{\text{Weight of nanoparticles}}\, \times \,100$$

### Morphological analysis

The morphology of the Cur-P-NPs was observed by transmission electron microscopy (TEM) on a JEOL JEM-1010 at 40,000× magnification.

### X-ray diffraction (XRD)

For the study of the surface properties of Cur-P-NPs, X-ray diffraction (XRD) analysis was carried out with the following parameters: output voltage = 40 kV, output current = 40 mA, and wave length = 0.1546 nm.

### In vitro stability of mPEG-Peptide-PCL and Cur-P-NPs

The mPEG-Peptide-PCL (100 mg) was dissolved in 10 mL tetrahydrofuran. Then, 2 mL of the mPEG-Peptide-PCL solution was diluted four-fold using 0.1 M PBS (pH 7.4), DMEM, and fetal bovine serum and incubated at 37 °C. At every given time point (0, 1, 4, 8, and 24 h), 1 mL of the sample solution was collected (for fetal bovine serum sample, excess acetonitrile was added to remove the protein, followed by centrifugation), and the Mn, Mw, and PDI of mPEG-Peptide-PCL were measured using GPC as described above.

Cur-P-NPs solution (2 mL) was diluted four-fold using 0.1 M PBS (pH 7.4), DMEM, and fetal bovine serum and incubated at 37 °C. At every given time point (0, 1, 4, 8 and 24 h), 1 mL of the sample solution was collected (for fetal bovine serum sample, excess acetonitrile was added to remove the protein, followed by centrifugation), and the particle diameter and PDI of Cur-P-NPs were measured.

### In vitro drug release

In vitro drug release was studied by dialysis. Cur-DMSO (5 mL) and Cur-P-NPs (5 mL × 2) with the same Cur content (150 μg/mL) were prepared. Dialysis of Cur-DMSO, the control group, was carried out at pH 7.4, and that of Cur-P-NPs (5 mL × 2), the treatment group, was carried out at 7.4 and 6.5, simulating the conditions of systemic circulation and weakly acidic tumor environment,respectively. The solutions were poured into dialysis bags (Mw 14 kDa). Then, the bags were submerged in 50 mL PBS at the respective pH values, and placed in an incubator at 37.0 °C ± 0.5 °C with shaking at 100 rpm. At predetermined time points (0, 1, 2, 4, 6, 12, 24, 36, 48, 60, 72, and 96 h), 3.0 mL of external solution was removed and replaced with an equivalent volume of fresh dissolution medium. The Cur content was determined by ultraviolet spectrophotometry.

### Cell culture

L929 mouse embryonic fibroblasts and A549 lung carcinoma cells were maintained in DMEM with 10% (v/v) fetal calf serum, penicillin (100 μg/mL), and streptomycin (100 μg/mL), and incubated in a humid atmosphere at 37 °C with 5% CO_2_.

### In vitro cytotoxicity of NPs

The cytotoxicity of NPs was evaluated by the 3-(4,5-dimethylthiazol-2-yl)-2,5-diphenyltetrazolium bromide (MTT) assay using L929 mouse embryonic fibroblasts. Cells were seeded in 96-well plates at a density of 1 × 10^5^ cells/well. After 24 h of appropriate growth, various doses of sterilized blank NP or medium only (negative control) were added, and the cells were incubated for 48 h. All samples were prepared in triplicate. A 20 μL volume of MTT labeling reagent was added, and cells were cultured for 4 h at 37 °C. The absorbance was measured at 570 nm, and cell viability (%) was represented as the ratio of the absorbance of the test and negative control solutions.

### In vitro anticancer activity assay

The efficacy of Cur-P-NPs solution against lung cancer cell viability was assessed in A549 cell lines by MTT analysis. Cells were seeded in 96-well plates at a density of 1 × 10^5^ cells/well, and incubated with 100 μL of the different dilutions of Cur-P-NPs or medium only (negative control) for 48 h. A 20-μL volume of MTT labeling reagent was added, and cells were co-cultured for 4 h at 37 °C. All samples were prepared in triplicate. The cell viability (%) was determined as per the method described in “In vitro cytotoxicity of NPs” section.

### Cellular uptake

L929 (as non-target cells) and A549 cells (as target cells) were seeded in 96-well plates (10^5^ cells/well), respectively. Thereafter, 100 μL of Cur-DMSO, blank NPs, Cur-NPs (prepared using mPEG-PCL without peptide modification), Cur-P-NPs, or DMSO only (negative control) with Cur content of 50 μg/mL were co-cultured at 37 °C for 4 h. The medium was then discarded, and the cells were washed with PBS thrice. Cellular uptake was evaluated by fluorescence microscopy at 200× magnification (Eclipse Ti-S; Nikon, Tokyo, Japan).

### Endocytic mechanism of Cur-P-NPs

To further study the endocytic mechanism of Cur-P-NPs in A549 cells, chlorpromazine (an endocytosis inhibitor of clathrin-mediated endocytosis), cytochalasin D (an endocytosis inhibitor of macropinocytosis mediated endocytosis), or genistein (an endocytosis inhibitor of caveolae-mediated endocytosis) were used, with no treatment as control [34]. Firstly, cells were seeded in 12-well plates (10^5^ cells/well) and co-cultured at 37 °C for 1 h with 100 μL of the endocytosis inhibitors at concentrations of 1, 5, or 25 μM. The medium was removed and replaced with complete medium containing Cur-P-NPs (Cur:50 μg/mL) and different inhibitors for another 15 min. Thereafter, the medium was removed and the cells were washed twice with PBS solution. The cells were finally analyzed by flow cytometry (Beckman Coulter Cytoflex; Beckman, USA). All experiments were carried out in triplicate.

### Biodistribution studies

Pharmacokinetic distribution studies were performed in nude mice [35]. About 5 × 10^7^ A549 cells in 200 μL PBS were subcutaneously injected into the left hind flank of the mice. Once the tumors had reached ~ 100 mm^3^ in size (typically 2 weeks later), 0.2 mL of Cur-DMSO, and Cur-NPs and Cur-P-NPs with a Cur content of 50 μg/mL were injected in the tumor-bearing mice via the tail vein at a Cur dose of 1.5 mg/kg. For the imaging studies, mice were anesthetized at the prescribed time points (1 and 6 h), and the images were shot using a small animal imager (IVIS, Lumina XRMS III) with Ex = 488 nm and Em = 520 nm. All the samples were prepared in triplicate.

### Statistical analysis

Results were expressed as mean ± standard error of the mean. Differences between groups were examined for statistical significance with the Student’s t-test, and P-values < 0.05 were considered statistically significant.

## Results and discussion

### Tri-block polymer characterization

**Fig. 2:**
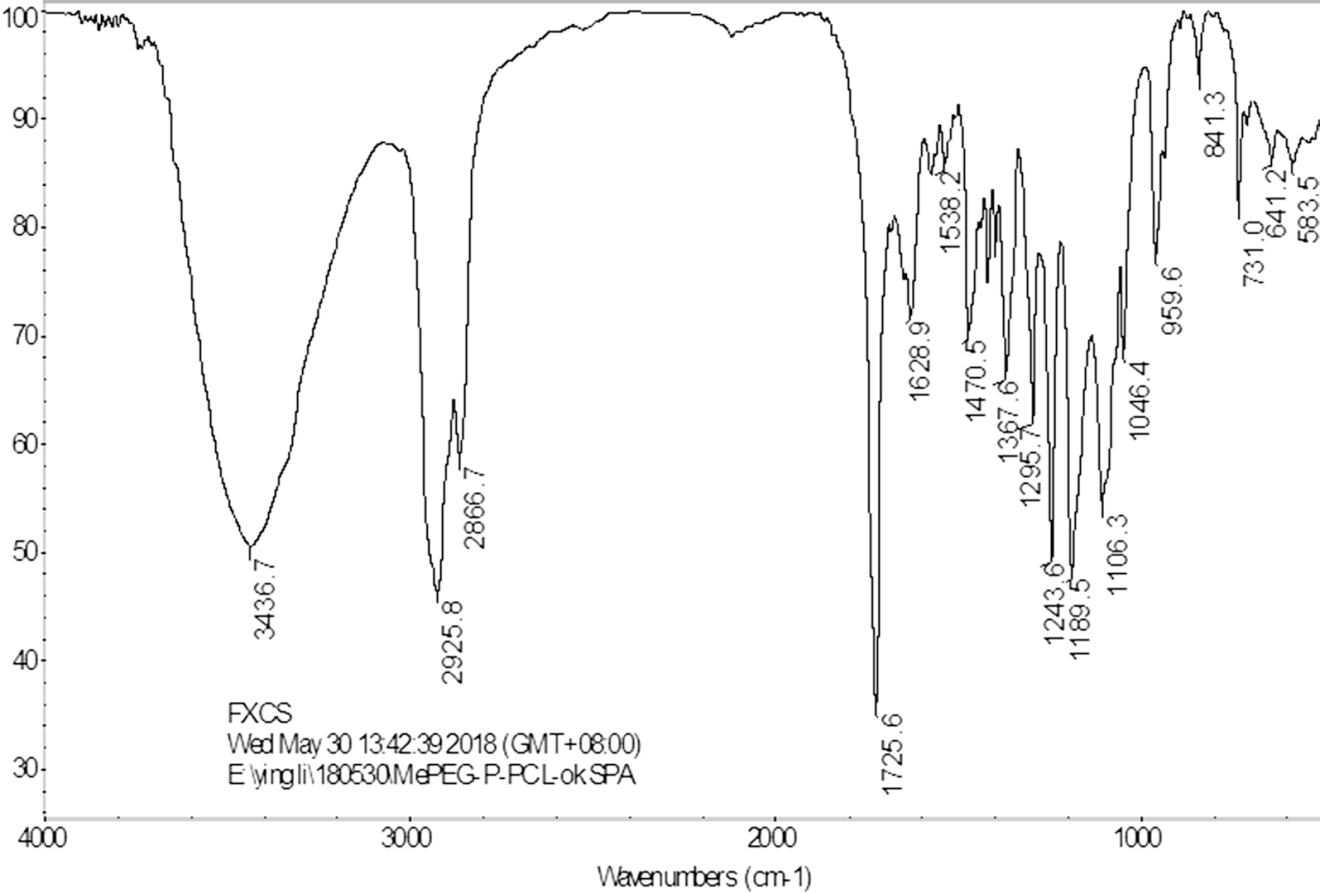
FT-IR spectra of mPEG-Peptide-PCL

**Fig. 3:**
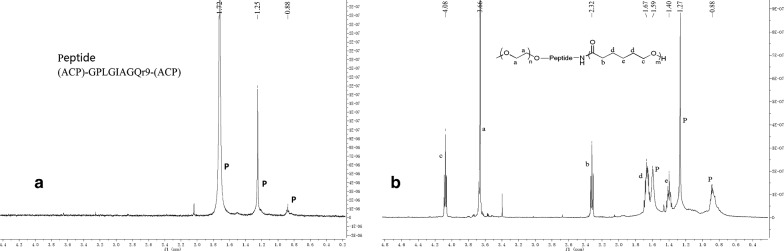
^1^H-NMR spectra of peptide (**a**) and mPEG-Peptide-PCL (**b**)

The polymer of mPEG-Peptide-PCL was characterized using FT-IR and ^1^H-NMR analyses. The FT-IR spectrum of mPEG-Peptide-PCL is presented in Fig. 2. Compared with that of mPEG-PCL, mPEG-Peptide and PCL-NH_2_ (Additional file 1: Figures S1–S3), the peak at 3436.7 cm^−1^ corresponds to the N–H bond stretching in the peptide linkage, while the peaks at 1628.9 and 1538.5 cm^−1^, which are attributed to the twisting vibrations of N–H bond, were observed very clearly. The bands at 2925.8 and 2866.7 cm^−1^ reflect the C–H stretching vibrations of the methylene group in PCL and mPEG, respectively. Additionally, the signals at 1725.0 and 1243.6 cm^−1^ (or 1189.5 cm^−1^) represent formation of the C=O and C–O–C bonds in the ester linkage. The corresponding ^1^H-NMR spectra and peak assignments are shown in Fig. 3a, b. The characteristic peaks of the peptide are showed at 1.72, 1.25 and 0.88 ppm, respectively (Fig. 3a). Compared to the peptide, the peak at 3.66 ppm is ascribed to the protons of the methylene group in mPEG, while the peaks at 4.08, 2.32, 1.67, and 1.40 ppm represent the methylene protons of PCL segments (Fig. 3b). All the above signals demonstrate the successful synthesis of the mPEG-Peptide-PCL.

Besides, the Mn, Mw, and PDI of the prepolymers measured by GPC were listed in Table 1. The molecular weight of mPEG-Peptide-PCL almost equaled the sum of those of PCL-NH_2_ and mPEG-Peptide, which indicated the regulation of synthesis. Meanwhile, mPEG-Peptide-PCL with the narrow PDI value was beneficial to the stability of the nanoparticles preparation.

**Table 1:** Mn, Mw, and PDI of the prepolymers

	Mn	Mw	PDI
PCL-NH_2_	4716	5666	1.20
mPEG-Peptide	6065	6259	1.03
mPEG-Peptide-PCL	9684	12,614	1.30

### Characterization of NPs

mPEG-Peptide-PCL polymer self-assembled to form biodegradation nanoparticles (Cur-P-NPs and blank P-NPs) with a hydrophilic shell (mPEG and peptide segment) and hydrophobic core (PCL segment). The particle sizes, zeta potentials, and EE and DL values are listed in Table 2. The average diameters of Cur-P-NPs and free P-NPs were 159.7 and 180.3 nm with PDI of 0.116 and 0.131, respectively, indicating a relatively narrow size distribution for Cur-P-NPs and free P-NPs. The zeta potentials ranged from 3.48 to 5.74 mV. The positively charged corona could prevent nanoparticle aggregation due to electrostatic repulsion, improving the cellular uptake efficiency. Moreover, the EE% and DL% values of Cur-P-NPs were 80.12 and 7.58%, respectively.

**Table 2:** Physicochemical characteristics of Cur-P-NPs

Sample	Particle size (nm)	PDI	Zeta potentials (mV)	EE%	DL%
Cur-P-NPs	159.7 ± 2.566	0.116 ± 0.017	5.74 ± 1.46	80.12	7.58
Blank P-NPs	180.3 ± 2.227	0.131 ± 0.025	3.48 ± 0.408	–	–

**Fig. 4:**
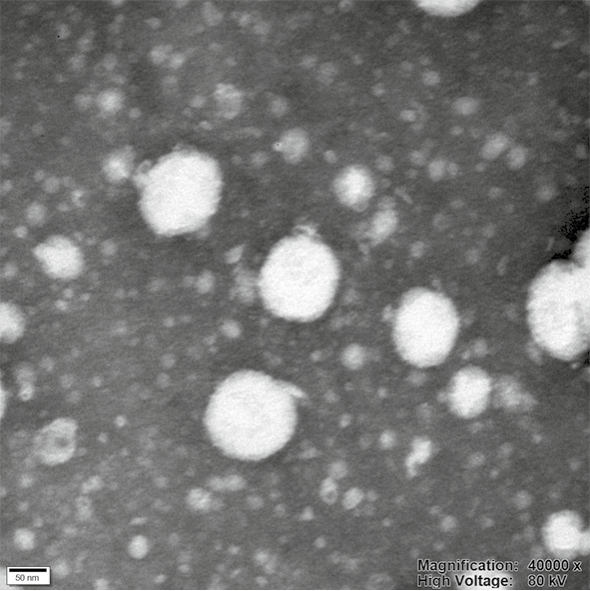
TEM photograph of Cur-P-NPs (under ×40,000 magnification)

According to the results observed in the electron micrographs (Fig. 4), the Cur-P-NPs possess a near-spherical shape, and were uniformly distributed in the suspension.

**Fig. 5:**
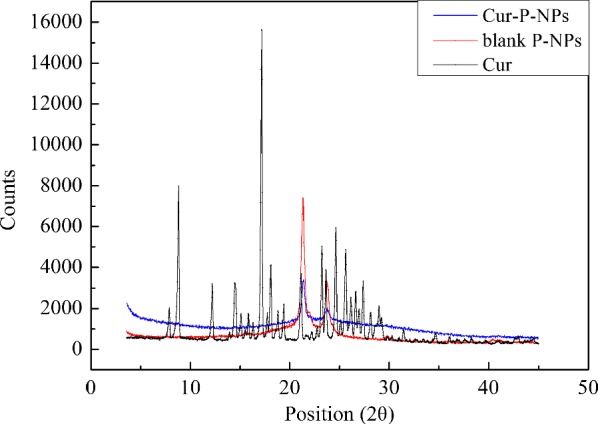
X-ray diffraction curve of Cur, Blank-NPs, and Cur-P-NPs

XRD analysis (Fig. 5) revealed the surface characteristics of native Cur, free P-NPs, and Cur-P-NPs. There are sharp, characteristic peaks in native Cur. However, these peaks disappeared in Cur-P-NPs, indicating that Cur was trapped in the NPs.

### In vitro stability of mPEG-Peptide-PCL and Cur-P-NPs

**Fig. 6:**
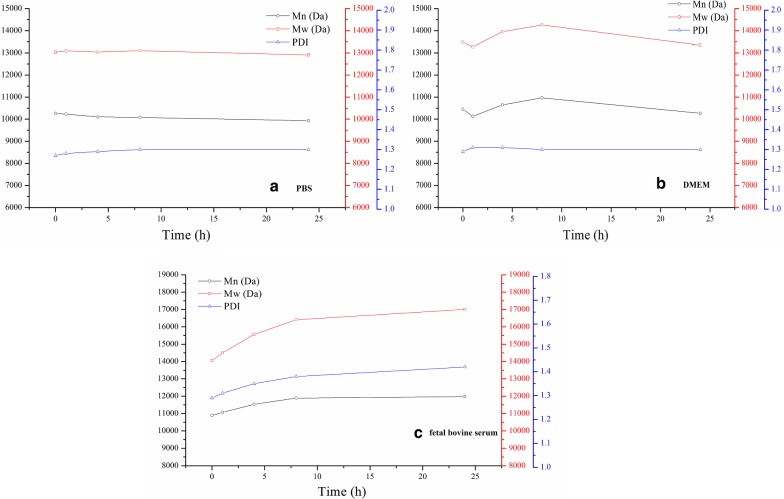
In vitro stability of mPEG-Peptide-PCL **a** PBS, **b** DMEM, **c** fetal calf serum

To preliminarily evaluate the validity of enzyme-responsive nanoparticles, the stability of mPEG-Peptide-PCL and Cur-P-NPs in PBS, DMEM, and fetal bovine serum were studied, respectively. mPEG-Peptide-PCL displayed excellent biostability in PBS and DMEM with Mn values changing to 300 and 700 Da, respectively (Fig. 6a, b). Both PDI were also stable at about 1.30. Moreover, the Mn of mPEG-Peptide-PCL increased to a minor extent to about 1000 Da (Fig. 6c) after incubation in fetal bovine serum, suggesting that some short chain polypeptide was adsorbed on the materials. For all the solutions, the Mn value of mPEG-Peptide-PCL did not show an obvious decrease [more than 1900; mPEG (Mn = 1900)], indicating that the hydrolysis of the peptide in mPEG-Peptide-PCL did not occur.

**Fig. 7:**
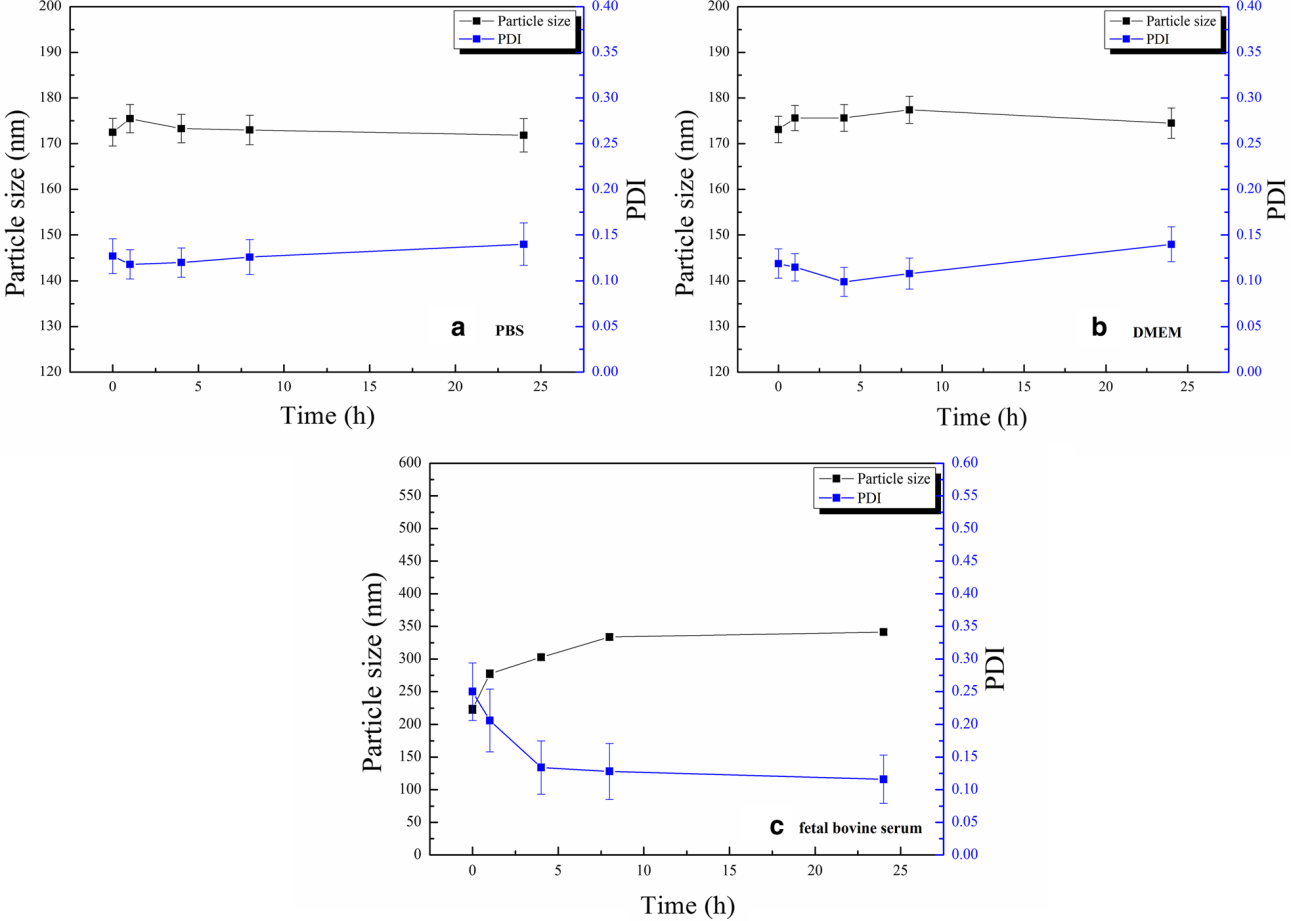
In vitro stability of Cur-P-NPs **a** PBS, **b** DMEM, **c** fetal calf serum

In addition, Cur-P-NPs displayed prominent stability in PBS and DMEM for 24 h with particle sizes of 170 and 175 nm, respectively (Fig. 7a, b). On the other hand, the protein adsorption effect was also observed in fetal bovine serum (Fig. 7c). The size of Cur-P-NPs increased in the first 8 h. Thereafter, a particle size of 335 nm was maintained. In particular, 8 h after incubation, the size of Cur-P-NPs was constant, suggesting that Cur-P-NPs could be generally stable in the body.

### In vitro drug release

**Fig. 8:**
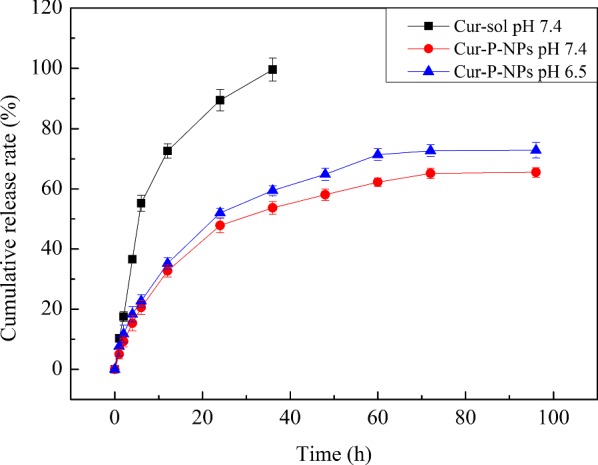
Accumulative release of Cur in vitro

Figure 8 shows the results of release kinetics of Cur from Cur-DMSO and Cur-P-NPs. On comparison of the release curves of Cur-DMSO and Cur-P-NPs at pH 7.4, Cur-P-NPs were found to show a lower release rate of Cur with 65.49% at 96 h than that of Cur-DMSO showing complete release at 36 h. Therefore, Cur exhibits a sustained release profile from Cur-P-NPs. Moreover, the Cur-P-NP solution in PBS were assessed at pH values of 7.4 and 6.5 to simulate the conditions of systemic circulation and weakly acidic tumor environment, respectively. The release curves almost overlapped in the first 6 h, thereafter, the final cumulative release rate of Cur reached 72.85% at pH 6.5, only exceeding the rate at pH 7.4 to a small extent. Similar in vitro release profiles of Cur from the NPs would guarantee a stable intracellular concentration of Cur, leading to a consistent therapeutic effect.

### Cytotoxicity analysis

The cytotoxicity of a material is one of the most important indicators for evaluation of biocompatibility of the delivery system. In this work, the viability of cells treated with blank P-NPs at different concentration is listed in Table 3. Cell viability decreased as the P-NP concentration increased from 0.51 to 2.05 mg/mL. In addition, on decreasing the P-NP concentration from 0.51 to 0.13 mg/mL, the cell viability was about 97%, and only a minor change in viability was observed. However, all cell viabilities were above 87.86%, indicating that blank P-NPs have good biocompatibility.

**Table 3:** Cytotoxicity evaluation of blank P-NPs for different concentration

Concentration (mg/mL)	Cell viability (%)
2.05 (origin solution)	87.86 ± 1.232
1.51	89.72 ± 1.077
1.02	93.46 ± 0.839
0.51	97.22 ± 0.955
0.25	97.96 ± 0.826
0.13	97.73 ± 0.426

### In vitro anticancer activity assay

**Fig. 9:**
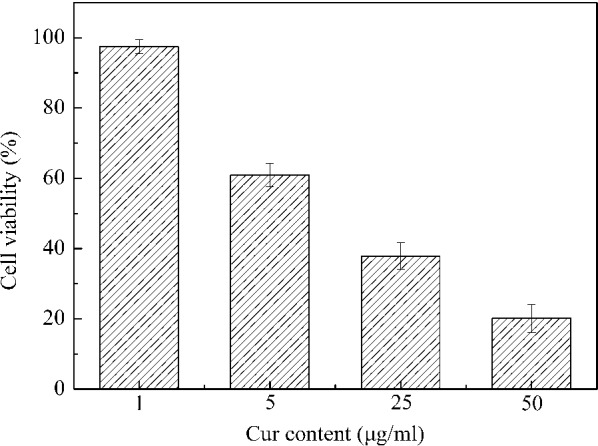
Pharmacodynamics of Cur-P-NPs at different concentrations

A study on the anticancer activity in vitro was performed by MTT analysis. The results could be a foundation for pharmacodynamics study in vivo, and have been summarized in Fig. 9. Cell viabilities of 97.46, 60.93, 37.87, and 20.15% were measured after 48 h of incubation with equivalent concentrations at 1, 5, 25, and 50 μg/mL, respectively. A dose-dependent anticancer activity was observed. However, any obviously anticancer activity was observed at low concentration (1 μg/mL), thus more than 5 μg/mL is the suggested concentration of Cur for efficient treatment of lung tumor.

### Cellular uptake

**Fig. 10:**
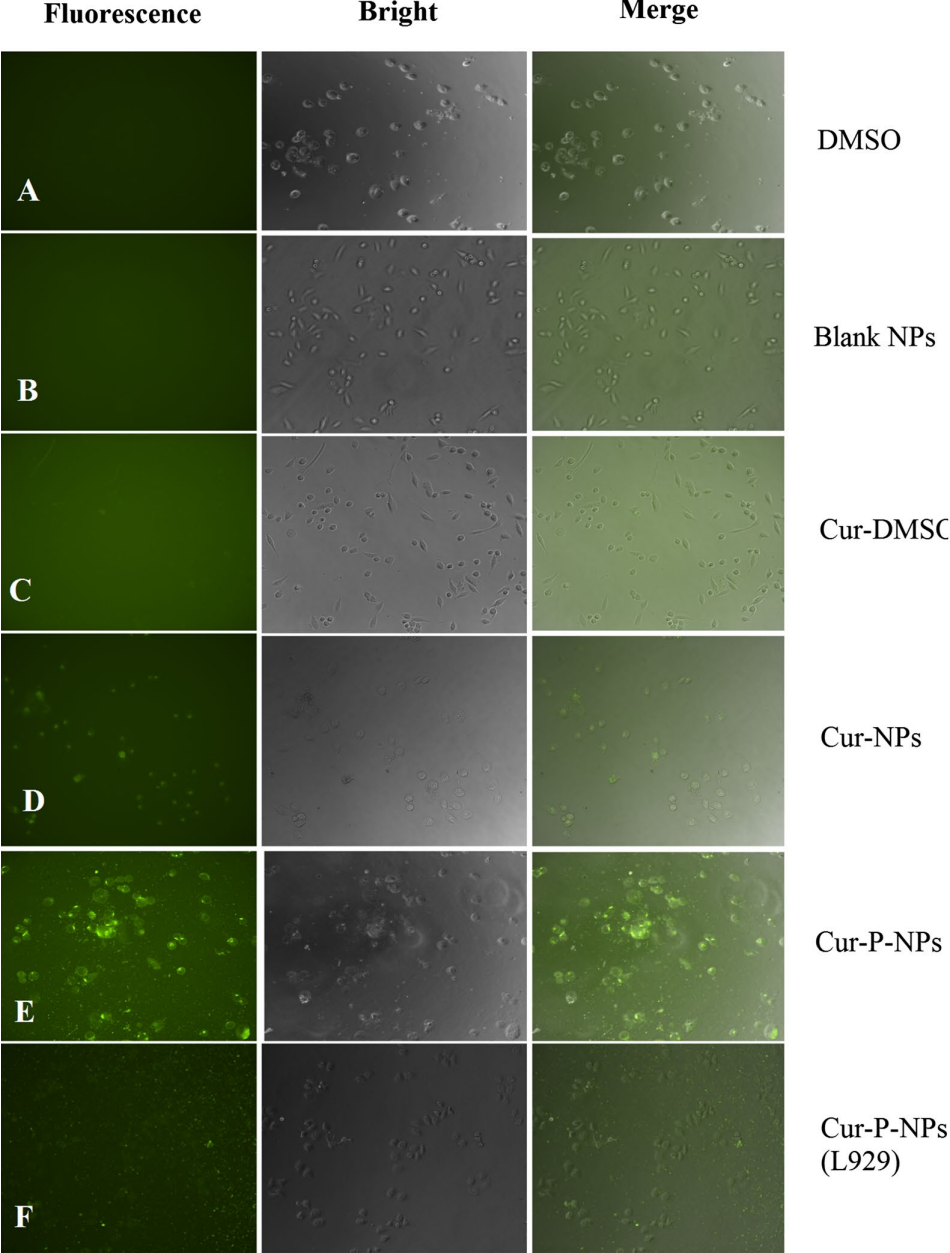
Cellular uptake in A549 and L929 cells. **A** DMSO, **B** blank-NPs, **C** Cur-DMSO, **D** Cur-NPs, **E** Cur-P-NPs, and **F** Cur-P-NPs(L929). (Under ×200 magnification)

The ability of cellular uptake and the enzyme-targeted availability for Cur-P-NPs were assessed by fluorescence microscopy (Fig. 10). There was no fluorescence associated with the negative control of DMSO or blank NPs (Fig. 10A, B). As shown in Fig. 10C–E, Cur-NPs and Cur-P-NPs showed a stronger fluorescence intensity than that of Cur-DMSO, demonstrating that the NPs had excellent permeability, which was consistent with the previous report [33]. On comparison with Fig. 10D, E, it was observed that Cur-P-NPs had stronger fluorescence intensity than Cur-NPs, indicating a higher cellular uptake of Cur-P-NPs. It was reported that PEGylation could hinder the cellular uptake ability, but in the present study, Cur-P-NPs showed excellent uptake ability. A reasonable explanation is that the ‘mPEG-GPLGIAGQ-r9’ was cut off to ‘mPEG-GPLGIAGQ’ and ‘r9-NPs’ by the MMP enzyme in A549, leaving ‘r9’ together with NPs so that its uptake ability was tremendously improved.

To further evaluate the enzyme-targeted availability of Cur-P-NPs, the ability of cellular uptake was compared between L929 (non-target cells) and A549 (target cells) cells. As illustrated in Fig. 10E, F, more Cur-P-NPs were internalized by A549 than L929, demonstrating that the Cur-P-NPs are a site-specific drug delivery system targeting tumors.

### Endocytic mechanism of Cur-P-NPs

**Fig. 11:**
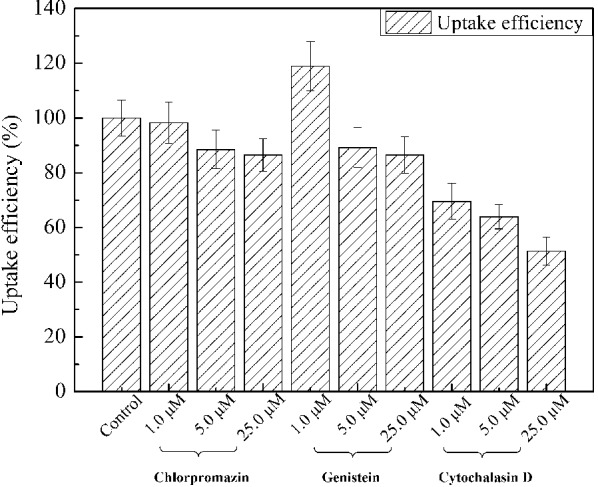
Endocytic mechanism of Cur-P-NPs

In this study, chlorpromazine (an endocytosis inhibitor of clathrin-mediated endocytosis), cytochalasin D (an endocytosis inhibitor of macropinocytosis mediated endocytosis), or genistein (an endocytosis inhibitor of caveolae-mediated endocytosis), as representative inhibitors with different inhibition mechanisms, were used to confirm the specific pathway for intracellular transport of mPEG-P-NPs. Results (Fig. 11) showed that cytochalasin D (macropinocytosis inhibitor) displayed the strongest inhibition with the uptake efficiency increasing from 51.31 to 69.54%, and the uptake efficiency was affected in a dose-dependent manner. Chlorpromazine and genistein (clathrin-mediated endocytosis and caveolae-mediated endocytosis) showed no significant inhibition at an uptake efficiency higher than 86.49%. These results highlighted that macropinocytosis was the main uptake mechanism for successful internalization of Cur-P-NPs into A549 cells.

### Biodistribution studies

**Fig. 12:**
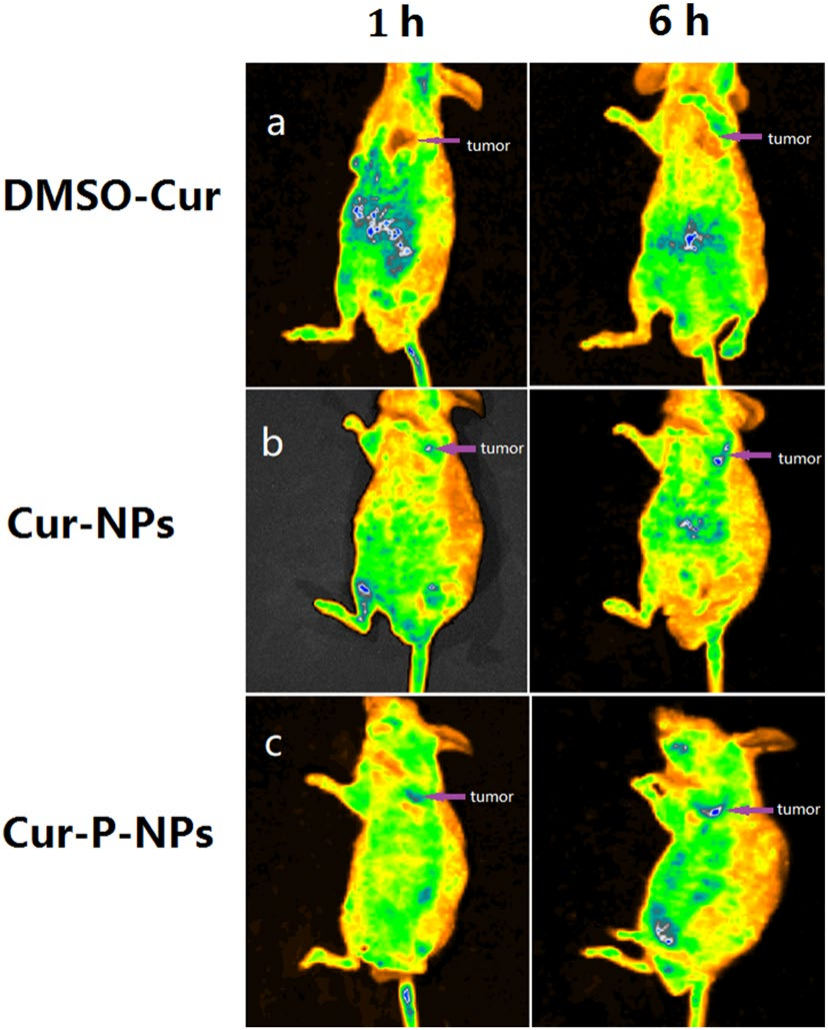
Biodistribution of Cur in vivo after i.v. injection **a** Cur-DMSO, **b** Cur-NPs, **c** Cur-P-NPs

To further test the targeting effect of Cur-P-NPs, Cur-DMSO and Cur-NPs (without peptide modification, prepared using mPEG-PCL) were used as the control groups. Real-time fluorescence images for in vivo Cur distribution are shown in Fig. 12. For Cur-DMSO, Cur was measured at very low levels in the tumor at 1 h; however, 6 h later, low fluorescence was observed in the tumor. Most of the Cur was found to accumulate in the abdomen, and the fluorescence effect became weak with time. Poor bioavailability and targeting by the Cur-DMSO resulted in simple passive diffusion and no drug release as well as rapid metabolism of Cur. For Cur-P-NPs and Cur-NPs, Cur-loaded nanoparticles enter cells mainly via active transport, and both nanoparticle preparations possess the property of sustained release. Therefore, a weaker fluorescence intensity was measured in the whole body compared to that of Cur-DMSO. With continuous release of Cur in vivo, brighter fluorescence is visible in the live animal and more Cur accumulation in the tumor (better tumor-targeting) was observed from 1 to 6 h after tail vein injection. Especially for Cur-P-NPs, brighter fluorescence is visible in the tumor after injection in 1 h, demonstrating that besides the EPR effect (passive targeting), the peptide of GPLGIAGQr9 carry out active targeting efficiently and enhance cellular uptake.

## Conclusions

In the current study, a novel mPEG-Peptide-PCL with enzyme sensitivity was successfully synthesized; the peptide can self-assemble to form nanoparticles as a biomimetic platform with tumor targeting ability. The cytotoxicity and stability analysis revealed that Cur-P-NPs exhibited excellent biocompatibility and biostability. The in vitro release profiles of Cur from the NPs were observed to be similar to those observed under systemic circulation and a weakly acidic tumor environment, indicating a stable intracellular concentration of Cur and a consistent therapeutic effect. The cellular uptake of drugs in tumor cells occurred mainly via macropinocytosis. Moreover, in nude mice, Cur-P-NPs displayed stronger fluorescence than Cur-DMSO and Cur-NPs, demonstrating more effective target efficiency and therapeutic effect. This result is in agreement with those reported by pharmacodynamics and cellular uptake studies in vitro. Therefore, Cur-P-NPs can be employed as an active targeting drug delivery system for lung cancer treatment.

## Additional file

**Additional file 1: Figure S1.** The FT-IR of mPEG-PCL. **Figure S2.** The FT-IR of mPEG-peptide. **Figure S3.** The FT-IR of PCL-NH_2_.

## Abbreviations

(ACP)-GPLGIAGQrrrrrrrrr-(ACP)
G: Gly
P: Pro
L: Leu
I: Ile
A: Ala
Q: Gln
r: d-Arg
