# Supplementary material for: PEGylated self-assembled enzyme-responsive nanoparticles for effective targeted therapy against lung tumors
Source: J Nanobiotechnology. 2018 Jul 16;16:57. doi: 10.1186/s12951-018-0384-8 (PMC6048871; doi:10.1186/s12951-018-0384-8)
Supplement: Supplementary file 1 — Additional file 1: Figure S1. The FT-IR of mPEG-PCL. Figure S2. The FT-IR of mPEG-peptide. Figure S3. The FT-IR of PCL-NH2. [file 12951_2018_384_MOESM1_ESM.docx]

**PEGylated self-assembled enzyme-responsive nanoparticles for effective targeted therapy against lung tumors**

Fangyuan Guo, Jiangqing Wu, Wenchao Wu, Dongxue Huang, Qinying Yan, Qingliang Yang, Ying Gao, Gensheng Yang*

*College of Pharmaceutical Science, Zhejiang University of Technology, Hangzhou 310014, China*

**Author list with email address:**

Fangyuan Guo guofy.2008@hotmail.com

Jiangqing Wu 751529082@qq.com

Wenchao Wu 1508445025@qq.com

Dongxue Huang 1178156772@qq.com

Qinying Yan yqy@zjut.edu.cn

Qingliang Yang qyang@zjut.edu.cn

Ying Gao 22357418@qq.com

**Corresponding author:**

Dr. Gensheng Yang

Postal address: College of Pharmaceutical Science, ZJUT

#18 Chaowang Road, Hangzhou, 310032, PR China

Tel: 86-571-88871077

Fax:86-571-88320913

Email: yanggs@zjut.edu.cn


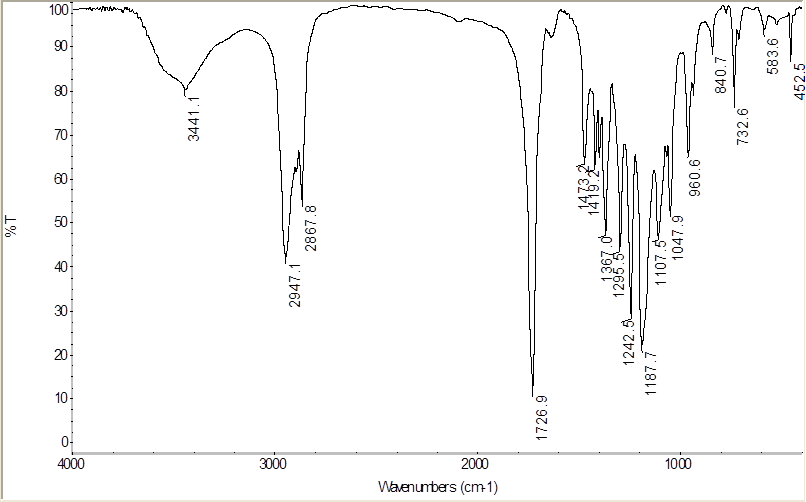


S1 The FT-IR of mPEG-PCL


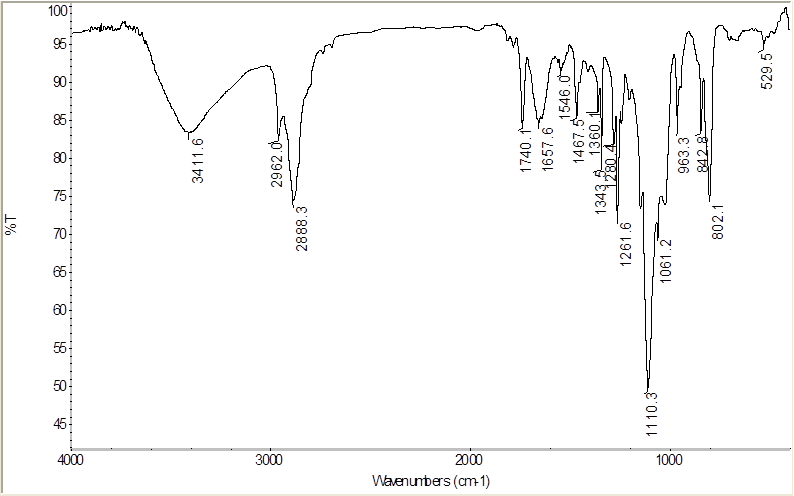


S2 The FT-IR of mPEG-Peptide


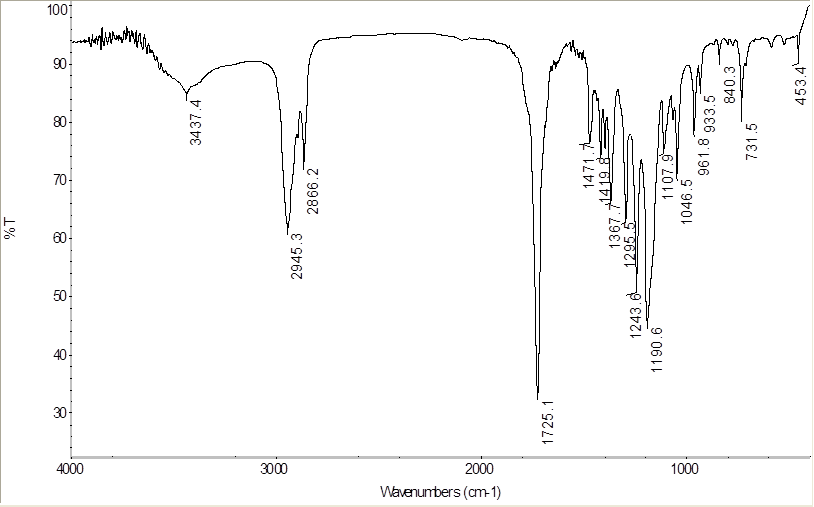


S3 The FT-IR of PCL-NH_2_
